# Sperm performance in conspecific and heterospecific female fluid

**DOI:** 10.1002/ece3.1977

**Published:** 2016-01-30

**Authors:** Emily R. A. Cramer, Even Stensrud, Gunnhild Marthinsen, Silje Hogner, Lars Erik Johannessen, Terje Laskemoen, Marie‐Christine Eybert, Tore Slagsvold, Jan T. Lifjeld, Arild Johnsen

**Affiliations:** ^1^ Natural History MuseumUniversity of OsloPO Box 1172Blindern0318OsloNorway; ^2^ EA7316Université de Rennes I263 Av. du Général Leclerc35042Rennes CedexFrance; ^3^ Department of BiosciencesCenter for Ecological and Evolutionary Synthesis (CEES)University of OsloPO Box 1066Blindern0316OsloNorway

**Keywords:** Cryptic female choice, postcopulatory prezygotic barriers, sexual selection, speciation, sperm competition, sperm motility, sperm velocity

## Abstract

Divergent sexual selection within allopatric populations may result in divergent sexual phenotypes, which can act as reproductive barriers between populations upon secondary contact. This hypothesis has been most tested on traits involved in precopulatory sexual selection, with less work focusing on traits that act after copulation and before fertilization (i.e., postcopulatory prezygotic traits), particularly in internally fertilizing vertebrates. However, postcopulatory sexual selection within species can also drive trait divergence, resulting in reduced performance of heterospecific sperm within the female reproductive tract. Such incompatibilities, arising as a by‐product of divergent postcopulatory sexual selection in allopatry, can represent reproductive barriers, analogous to species‐assortative mating preferences. Here, we tested for postcopulatory prezygotic reproductive barriers between three pairs of taxa with diverged sperm phenotypes and moderate‐to‐high opportunity for postcopulatory sexual selection (barn swallows *Hirundo rustica* versus sand martins *Riparia riparia*, two subspecies of bluethroats, *Luscinia svecica svecica* versus *L. s. namnetum,* and great tits *Parus major* versus blue tits *Cyanistes caeruleus*). We tested sperm swimming performance in fluid from the outer reproductive tract of females, because the greatest reduction in sperm number in birds occurs as sperm swim across the vagina. Contrary to our expectations, sperm swam equally well in fluid from conspecific and heterospecific females, suggesting that postcopulatory prezygotic barriers do not act between these taxon pairs, at this stage between copulation and fertilization. We therefore suggest that divergence in sperm phenotypes in allopatry is insufficient to cause widespread postcopulatory prezygotic barriers in the form of impaired sperm swimming performance in passerine birds.

## Introduction

Coevolution of sexual traits and sexual preferences within isolated populations can drive speciation, if evolution occurs in different directions within each population (Lande 1981; Coyne and Orr 2004). Under such circumstances, if the populations come into secondary contact, members of one population may not consider members of the opposite population attractive mating partners, resulting in a reproductive barrier between the groups (Lande 1981; Coyne and Orr 2004). Similarly, divergence in traits involved in ejaculate–female or ejaculate–egg interactions may result in reduced fertilization efficiency between members of different populations (Howard et al. 2009; Palumbi 2009). The hypothesis that strong divergent sexual selection during allopatry can, as a by‐product, lead to speciation has generated a large body of work (reviewed in Ritchie 2007; Palumbi 2009; Kraaijeveld et al. 2011). While the hypothesis remains contentious (Ritchie 2007; Kraaijeveld et al. 2011), it is supported by several lines of evidence. Divergence in sexual phenotypes is faster in taxa with greater opportunity for sexual selection (Dorus et al. 2004; Price and Whalen 2009; Ramm et al. 2009; Seddon et al. 2013; Rowe et al. 2015), and divergence in sexual phenotypes (e.g., visual and acoustic signals, sperm morphology, ejaculate proteins) is often higher than divergence in nonsexual traits (Mendelson and Shaw 2005; Ritchie 2007; Pitnick et al. 2009; Safran et al. 2012; Martin and Mendelson 2014). Furthermore, in several taxa, populations with more diverged sexual phenotypes are less likely to interbreed, an important assumption of this hypothesis (Ryan and Rand 1995; Mendelson and Shaw 2002; Nosil et al. 2002; Zigler et al. 2005; Ortiz‐Dominguez et al. 2006; Stelkens and Seehausen 2009; Grace and Shaw 2011; Willis et al. 2014). While the hypothesis that divergent sexual selection can lead to reproductive barriers between species has been tested from a number of perspectives and in many taxa, it remains relatively untested in the context of postcopulatory prezygotic barriers acting in internally fertilizing vertebrates.

Relatively little is known in general about postcopulatory prezygotic barriers in internally fertilizing vertebrates. In birds, such barriers could arise at several stages between copulation and fertilization (Birkhead and Brillard 2007; Cramer et al. 2014). First, the vast majority of inseminated sperm fail to swim across the vagina, and the mechanism of this reduction in sperm numbers has been linked to the specific proteins expressed on the surface of the sperm cells (Steele and Wishart 1992, 1996). Barriers at this stage appear likely to arise because of species differences in vaginal chemistry, including the complement of female proteins and glycoproteins (although no data are available in birds, these affect sperm–female interactions within species in mammals, e.g., Tollner et al. 2012), and the concentration of various ions, which can have species‐specific effects (birds: Wishart and Wilson 1999; fish: Beirão et al. 2014). After crossing the vagina, species‐specific barriers could also occur while sperm are stored in the female's sperm storage tubules and/or when sperm interact with the ovum at the point of fertilization, although current evidence suggests these barriers may be relatively weak compared to the vaginal barrier (Bakst et al. 1994; Stewart et al. 2004; Sellier et al. 2005; Birkhead and Brillard 2007).

Here, we test for postcopulatory prezygotic barriers within three pairs of passerine bird taxa, to test the hypothesis that such barriers arise due to divergent selection within allopatric populations or species. We focus on three pairs of taxa that may be particularly likely to show such barriers, because they have divergent sperm morphology and moderate‐to‐high sperm competition. We use divergence in sperm morphology as a likely indicator of overall divergence in sperm phenotype and biology, although other characteristics, such as the suite of sperm surface proteins, may be the primary mechanism of postcopulatory prezygotic barriers (Steele and Wishart 1992, 1996). Sperm competition within species generates the opportunity for postcopulatory sexual selection to act, and postcopulatory sexual selection appears to have had a strong influence on sperm morphology and swimming speed across passerine species (Calhim et al. 2007; Immler and Birkhead 2007; Kleven et al. 2008, 2009; Lüpold et al. 2009a, 2009b; Lifjeld et al. 2010).

We evaluated the presence of postcopulatory prezygotic barriers by testing sperm swimming speed and the proportion of motile sperm cells in fluid from the distal female reproductive tract of conspecific and heterospecific females (Cramer et al. 2014). This experimental approach was used because sperm are likely to be under the strongest selection as they swim across the vagina (Steele and Wishart 1992; Bakst et al. 1994), and swimming speed and the proportion of motile cells are important metrics of sperm performance in many taxa (Simmons and Fitzpatrick 2012), including passerines (Birkhead et al. 1999; Kleven et al. 2009; Bennison et al. 2014; see also Lüpold et al. 2009a; Laskemoen et al. 2010). In the presence of a postcopulatory prezygotic barrier, we predict that sperm swimming speed and/or the proportion of motile sperm would be reduced in heterospecific female fluid compared to conspecific female fluid and control fluid. We test this prediction in three reciprocal crosses: between two species of swallows (barn swallows *Hirundo rustica* and sand martins *Riparia riparia*), between two subspecies of bluethroats (*Luscinia s. svecica* and *L. s. namnetum*), and between two species of tits (great tits *Parus major* and blue tits *Cyanistes caeruleus*). For ease of reference, we use “conspecific” and “heterospecific” to include members of the same and opposite subspecies, as well as full species. We further validate our experimental protocol by testing that the female fluid samples contain proteins.

These species are ideal for testing whether postcopulatory prezygotic barriers evolve in passerines via divergent postcopulatory sexual selection during allopatry, because they have characteristics that make such barriers likely under this hypothesis. Specifically, sperm morphology differs between the two members of each pair of taxa, likely indicating that sperm phenotypes differ dramatically enough for cryptic female choice to discriminate among sperm from the different species (Table 1). Bluethroats, in particular, have exceptionally rapid rates of sperm evolution (Hogner et al. 2013). Due to the moderate‐to‐high levels of extrapair paternity within each taxon (Table 1), there is high opportunity for postcopulatory sexual selection, such that mechanisms of female choice for sperm traits may be have evolved via within‐population processes in each taxon. Because these species do not commonly hybridize in the wild (Table 1), a detected postcopulatory prezygotic barrier could be attributed to divergence in phenotypes during isolation, rather than ongoing reinforcement acting on sperm phenotypes after secondary contact (e.g., Lorch and Servedio 2007; Matute 2010). Finally, because this set of species encompasses substantial taxonomic breadth, as well as including both recently diverged and more anciently diverged lineages (Table 1), our results should provide general insights into the occurrence of postcopulatory prezygotic barriers in passerines, at various stages in the evolutionary diversification of species.

**Table 1:** Background information on species pairs used in experiments. We estimated divergence between species based on sequence divergence in mitochondrial cytochrome *c* oxidase I (COI). Information on the occurrence of hybridization and on the frequency of extrapair young (EPY) in the wild is also given. EPY and total sperm length (TSL) estimates were from the study populations examined in this study, or nearby populations

Species pair	COI divergence (%)^1^	Natural hybridization?	TSL divergence (%)^2^	Species	% nests with EPY	TSL (mean ± SD, *μ*m)
Swallows	15.3^3^	One documented hybrid^4^	30.3	Barn swallow	50^5^	90.6 ± 2.4^7^
				Sand martin	37^6^	123.0 ± 4.5^7^
Bluethroats	0.3^8^	Unknown; subspecies are allopatric	2.7	Bluethroat (ssp. *namnetum*)	64^9^	206.1 ± 5.4^11^
				Bluethroat (ssp*. svecica*)	49.5^10^	211.8 ± 5.6^11^
Tits	10.2^3^	No natural hybrids documented^12^	6.4	Great tit	31^13^	98.0 ± 3.4^7^
				Blue tit	29^13^	104.8 ± 2.6^7^

^1^Calculated as the Kimura 2 parameter; ^2^standardized divergence calculated as the difference between lengths, divided by the mean length between species; ^3^data from the International Barcode of Life database; ^4^Heneberg (1997), McCarthy (2006); ^5^Kleven et al. (2006); ^6^Augustin et al. (2006); ^7^TL and JTL, unpublished; also see Lifjeld et al. (2010); ^8^SH, AJ et al. unpublished; ^9^Questiau et al. (1999); ^10^Johnsen and Lifjeld (2003); ^11^Dobbe (2014); ^12^Slagsvold et al. (2002); ^13^Johannessen et al. (2005).

## Methods

### Study sites and field procedures

#### Swallows

Barn swallows were captured with mist nets in a barn near Fredrikstad, Norway (59°09′N, 11°05′E) on 17–18 June 2013 and 17–18 June 2014. It was not possible to evaluate nesting stage directly, but several females had well developed brood patches, and some nests were being provisioned by adults, indicating that a few had hatched. Barn swallows in Norway frequently produce multiple broods within a breeding season and breed relatively asynchronously (Haftorn 1971), so it is likely that some females' reproductive tracts were physiologically prepared for sperm choice, although females with nestlings appear unlikely to be actively choosing sperm.

Sand martins were caught with mist nets at a sand pit at dawn, near Sarpsborg, Norway (59°17′N, 11°02′E), on 18 June 2013 and 2014. Three of the females sampled in 2014 appeared to have fully developed eggs in their abdomens, ready to be laid that morning.

#### Bluethroats

The *svecica* subspecies was studied at Øvre Heimdalen, Norway (61°25′N, 8°52′E, Øystre Slidre, Oppland), in the following periods: 6–16 June 2012, 29 May–7 June 2013, 29 May–3 June 2014, and 29 May–4 June 2015. We located nests in this population, and first egg dates were 28 May–11 June in 2013 and 2014; in 2015, the majority of first egg dates were estimated between 7 and 13 June.

The *namnetum* subspecies was studied at several locations in Brittany, France (Brière marsh, 47°21′N, 2°13′W; Guérande salt pans, 47°17′N, 2°28′W; and Marais du Mès salt pans, 47°24′N, 2°24′W), in the periods 7–17 April 2013, 8–13 April 2014, and 13–19 April 2015. This period roughly corresponds to the nest‐building and preincubation stages, based on the following observations. Most males were singing and performing displays at the beginning of the field sessions, and many had become less conspicuous, presumably beginning to mate guard, by the end of the field session. No females had evidence of brood patch formation at the time of capture, several females were seen carrying nesting material, and the two nests found with eggs during this period had a first egg date of 8 April 2014.

Males and some females were captured using mist nets or song post traps with playback of conspecific song, or using food‐baited ground traps. Additional *svecica* females were captured at their nest sites, either early in incubation (2012) or on the 6th day of egg laying (2013).

#### Tits

Tits of both species were captured at two field sites near Oslo, Norway (Brenna: 60°01′N, 10°37′E; and Dæli: 59°56′N, 10°33′E), between 28 April and 26 May 2015. Most blue tit females were captured on 28 and 29 April, with 1–7 eggs laid in their nests, and most great tit females were captured between 5 and 13 May, with 4–8 eggs (and one female nest‐building, apparently renesting). As copulations appear to occur throughout the laying sequence for blue tits (Johnsen et al. 2012), and great tits still have a relatively large number of stored sperm throughout egg laying (Lifjeld et al. 2000), this appears to be the optimal time to sample females for sperm selection. All experiments were conducted on males at Brenna, between 21 and 26 May. Only two great tit females and one blue tit female were captured at Brenna, so most females were unexposed with the individual males used in the experiments. All birds were captured with mist nets using playback and live, caged tits as lures.

#### General

All birds were released immediately upon completing sampling. Ethical permissions for fieldwork were to AJ (license 2014/53673 from the Norwegian Animal Research Authority, ringing license 680 from the Norwegian Environment Agency), ERAC (ringing license 1352 from the Norwegian Environment Agency), MCE (authorization for animal experimentation from the prefecture of Ille et Vilaine, number 35‐04, and ringing permit 1314 from the French National History Museum), and TS (license 7390, 2015/30725‐1 from the Norwegian Animal Research Authority, ringing license 2014/2620 from the Norwegian Environment Agency).

### Experimental protocol

**Figure 1:**
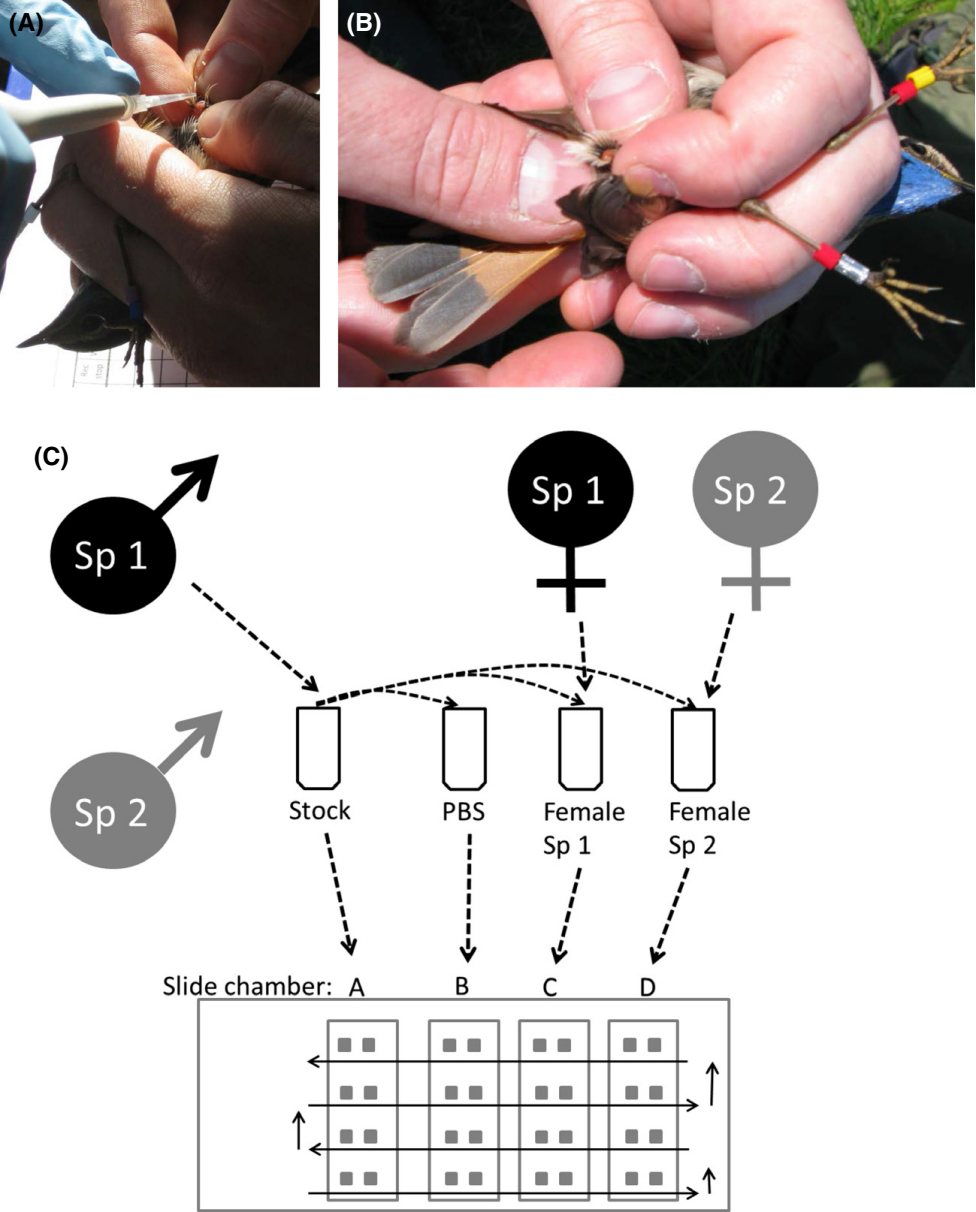
Summary of methods. (A) The female reproductive tract was washed with a phosphate‐buffered saline (PBS) solution. These samples were frozen for later use. (B) Ejaculates were collected via cloacal massage. (C) Sperm and thawed (prewarmed) female fluids were mixed, as follows. The ejaculate was diluted in PBS to create a stock solution; 2.5 *μ*L of this stock solution was added to each of three experimental treatments (control PBS; a conspecific female fluid, and a heterospecific female fluid). Treatments were loaded onto a four‐chambered microscope slide and filmed (small gray boxes and solid arrows, showing eight filming locations). As per Appendix 1, the number of filming locations varied among species pairs. The stock solution was not filmed in tit experiments. The assignment of female species to chamber was rotated among blocks. Sp = species

We collected samples from the female reproductive tract as follows. First, we swabbed the exterior of a female's cloaca with 96% ethanol and allowed it to air‐dry (except for *svecica* females in 2012, which were not cleaned first). We then massaged the cloaca to evert the inner mucosa and pipetted a small volume (2–5 *μ*L) of sterile phosphate‐buffered saline (PBS) onto the exposed surface. After waiting 5 sec for equilibration, we collected the droplet of PBS into a skirted microcentrifuge tube (Fig. 1A). We repeated this process until we had collected 15 *μ*L of fluid, or until we had used a maximum of 45 *μ*L PBS in our attempts. To obtain homogenous subsamples that could be used independently from each other, we mixed the collected fluid by pipetting and divided it into three microcentrifuge tubes, each containing 5 *μ*L of female fluid. Samples from *svecica* females in 2012 were stored in two clay‐capped capillary tubes. Female fluids were frozen immediately after collection and were kept frozen until immediately before use. Sand martin female fluids, as well as some bluethroat samples, were used on the same day that they were sampled, but they were frozen for at least 30 min before use to ensure that all samples had been through one freeze‐thaw cycle.

Upon catching a male, we collected a sperm sample via cloacal massage (Fig. 1B, Wolfson 1952). To dilute the sample to an appropriate working concentration, and to obtain sufficient volume for the experiments, we transferred this sperm sample into prewarmed (to 40°C) PBS (12 *μ*L for bluethroats, tits, and swallows in 2014; 20 *μ*L for swallows in 2013). Two *μ*L of this stock solution were added to one well of a four‐chamber slide to check for motile sperm, and additional prewarmed PBS was added to the stock tube if sperm concentration was too high for optimal video analysis. If the ejaculate was of high quality, we added 2 *μ*L of the stock solution to each of three treatment tubes containing 5 *μ*L of PBS, conspecific female fluid, or heterospecific female fluid (Fig. 1C). Fifteen bluethroat experiments and 10 swallow experiments were run at half‐volume because of logistical problems (i.e., 1 *μ*L sperm mixture added to 2.5 *μ*L of the experimental fluids). After adding the ejaculate, we put 2.5 *μ*L of each mixture onto a separate chamber of the same four‐chamber slide, and video‐recorded sperm swimming in each of the four chambers (three experimental and one stock) consecutively. Each chamber was filmed 3–8 times (depending on species and year; see “Appendix” for details), with each filming lasting at least 0.5 sec, and different filmings taken in different locations on the slide to avoid recording individual sperm multiple times. Experiments were performed in “blocks” consisting of four individuals (one of each sex and each species). We maintained the assignment of female species to slide chambers within blocks of individuals, such that for each group of four individuals (one female of each species and one male of each species), an individual female's fluid was in the same slide chamber for each experiment. This assignment was rotated among experimental blocks. All solutions and slides were prewarmed to 40°C before the experiment began. The stock concentration was filmed for all experiments except for *namnetum* experiments in 2013 and tit experiments, but we excluded stock data in these analyses to avoid potential confounds due to differing sperm concentrations. We use “experiment” to refer to the set of recordings made from a single ejaculate.

We modified the magnification settings and temperature control device between years in order to maximize the sample size of sperm cells filmed while obtaining high enough resolution images to analyze (see “Appendix” and details on sample size criteria used in analysis, below). Within a single experiment, though, all treatments experienced the same conditions, and we accounted for such variation statistically by including “year” as a random term.

### Video analysis

We analyzed videos using computer‐assisted sperm analysis (Hamilton Thorne Research, CEROS software, Beverly, MA). In order to exclude poorly tracked sperm cells from analyses, we applied several filters based on CEROS's quantification of sperm motion. Variation in the filter settings used across species and years was due to differences in video quality, sperm concentration, and sperm behavior; in all cases, we used thresholds that excluded most of the poorly tracked cells without also excluding a large number of well‐tracked cells. For tracked cells used in analyses of sperm velocity, we applied the following filters: straightness (STR) > 80, linearity (LIN) > 60, elongation < 50 (bluethroats and tits only), ≥10 points in the detection series, and 0 gaps in the detection series. For swallow videos in 2014, we used STR > 90 and LIN > 65, and for tit videos, we used STR > 90 and LIN > 60. For bluethroats and tits, we also removed from swimming speed analysis tracks where a single movement between successive cell detections was >5 times the interquartile range of movements for that track. Curvilinear velocity (VCL) was used as the estimate of sperm swimming speed, following the logic of Laskemoen et al. (2010); VCL was strongly correlated with both VAP and VSL in all datasets (*F* > 9674, *P* < 0.0001). Tracks with smoothed‐path velocity (VAP) <30 or straight‐line velocity (VSL) <25 were moving due to drift and were considered static (i.e., not included in sperm swimming speed analyses). We calculated the proportion of motile cells, including these drifting cells and truly static cells as static, and including all moving cells as motile. For bluethroats in 2014 and 2015 and for the tits, moving tracks with elongation >50 were nonsperm contaminants and were excluded from the dataset completely.

### Protein analysis

We tested protein concentrations of female fluid samples using BioRad Experion Pro260 microfluidic chips (Hercules, CA). These chips separate proteins with different molecular weights and migration properties and quantify total protein content at each molecular weight. Our main goals were to ascertain that our sampling protocol was effective at collecting proteins and, because female fluid samples were stored over a relatively long time period for bluethroats, to test for changes during storage. Protein analyses for swallows and bluethroats were run on 1–2 October 2014 or on 3 February 2015, and for tits on 21 August 2015. All analyses were run under reducing conditions with mercaptoethanol as the reducing agent, following the manufacturer's instructions. Each chip contained up to 10 samples. To best compare between taxa within a pair and between storage durations within a taxon, we ensured that chips contained samples collected from each year (swallows and bluethroats), and from each taxon within a pair.

### Statistical analysis

Preliminary analyses suggested that sperm swimming speed and the proportion of motile cells changed over time within experiments. We therefore include time (seconds from the beginning of filming, divided by 60 to facilitate analysis) in our models. To ensure adequate coverage over time for each treatment, for models of VCL, we included only experiments with at least three well‐tracked (according to the above criteria) moving cells in both the first and the second half of the filmings, for that treatment. For the proportion of motile cells, we include only experiments with at least eight cells detected in both the first and the second halves of the filmings. For experiments on swallows in 2013, there were three filmings per treatment, and we applied the sample size cutoffs to the first and last filmings. For analyses of VCL, we used individual cells as the unit of analysis (averaging across cells is undesirable as it ignores informative variability within ejaculates and variation in the number of cells tracked per male; Amann and Waberski 2014). For proportion motile, we used the proportion of motile cells in each filming location as the dependent variable. We avoided pseudoreplication using random effect terms, detailed below.

To test fixed effects, we began with a three‐way interaction term between male species, treatment (i.e., control, or the species of the female) and time, as well as the constituent pairwise interactions and main effects. We then removed nonsignificant interactions in a backwards stepwise procedure, beginning with the highest‐order interactions; we report only the simplified version of the models. Models contained a random intercept for male, female, male–female combination, and year (the latter for swallows and bluethroats only, as tit experiments were conducted only in 1 year). To improve model function, the control treatment was assigned a different random “identity” for each experimental block. In this framework, a postcopulatory prezygotic barrier may be supported if there is a significant three‐way interaction between male species, treatment, and time (with sperm swimming performance changing differently over time depending on the combination of species considered) or if there is a significant two‐way interaction between male species and treatment (with sperm swimming performance being different on average, depending on the specific combination of male and female).

To compare protein content between species within pairs, we constructed a separate model for each species pair, using either log‐transformed total protein concentration or the number of protein peaks detected as the response variable, year and female species as fixed factors, and analysis chip as a random effect. In this context, the year effect tests the effect of storage duration, as samples were collected in different years but analyzed simultaneously.

We used the package lme4 (Bates et al. 2015) with significance estimated via lmerTest (Kuznetsova et al. 2014). For post hoc testing, we reran models and iteratively changed the reference level for variables that were in significant interactions or that had significant main effects, and corrected for multiple testing using false discovery rate correction. Graphs were constructed using ggplot2 (Wickham 2009). All analysis was conducted in R 3.0.3 (R Development Core Team, Vienna, Austria), with model assumptions validated by eye, following Zuur et al. (2009).

## Results

### Swallows

**Figure 2:**
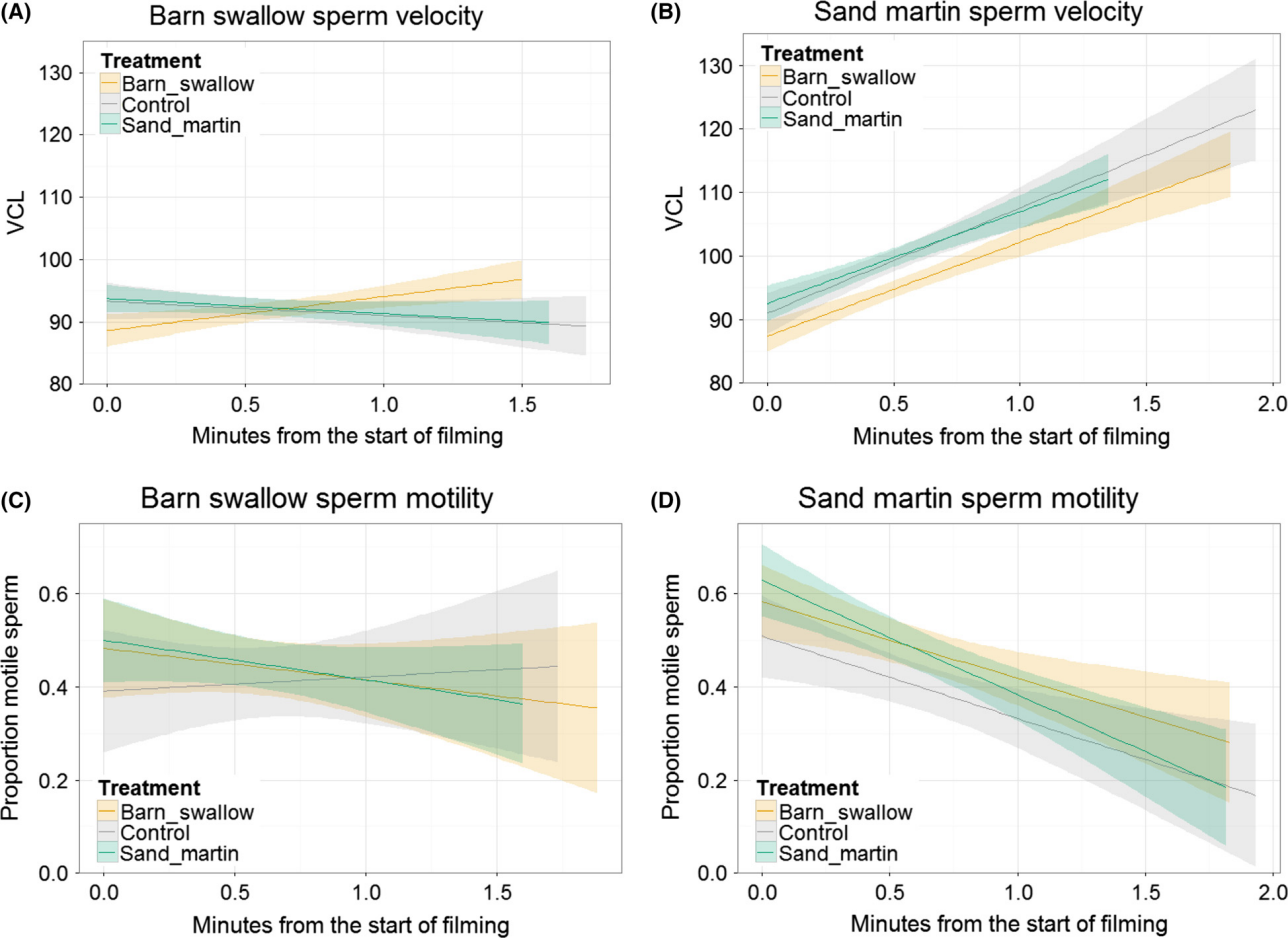
Change in sperm swimming parameters (A, B: sperm velocity, VCL,* μ*m/S; C, D: proportion of motile sperm) over time in barn swallows (A, C) and sand martins (B, D). Sperm were mixed with fluid from the reproductive tract of barn swallow females (tan), sand martin females (blue) or a control saline solution (gray) before being filmed. (A, B) Sand martin sperm swimming speed increased over time more than did barn swallow sperm swimming speed, and barn swallow female fluid supported a faster increase in sperm swimming speed for males of both species. (C, D) The proportion of motile cells decreased over time, and was lower in the control treatment than in female fluids, for both male species. Decrease over time was faster in sand martin ejaculates than in barn swallow ejaculates. Plots drawn using ggplot2 using raw data; shading indicates 95% confidence intervals; statistical tests were performed with both male species considered simultaneously, although they are drawn separately here.

Sperm swimming speed changed over time in swallows, with changes depending on both male species and treatment (Fig. 2A,B; Table 2). Sand martin sperm swimming speed increased over time relative to barn swallow sperm (estimated difference in change over time 4.89 ± 1.69). Barn swallow female fluid caused sperm swimming speed from males of both species to increase more over time relative to sand martin female fluid (difference in slope 4.21 ± 1.81, *t*_4053_ = 2.33, adjusted *P* = 0.04) and relative to control fluid (4.43 ± 2.02, *t*_3723_ = 2.19, adjusted *P* = 0.04). However, barn swallow female fluid also tended to cause a decrease in the initial swimming speed of sperm from both species (i.e., the intercept of the line relating VCL to time), although no pairwise differences among intercepts were significant after correction for multiple testing (adjusted *P* > 0.14). There were no significant differences in VCL between sperm in sand martin female fluid and in control fluid. Interaction terms that were predicted in the presence of a postcopulatory prezygotic barrier were nonsignificant and removed from the reported model (male species × treatment × time, *F*_2,4148.7_ = 1.12, *P* = 0.33; male species × treatment, *F*_2,213.6_ = 0.51, *P* = 0.60).

**Table 2:** Reduced model relating sperm velocity to treatments and time in swallows, after removing nonsignificant interaction terms. Post hoc comparisons between treatment groups are reported in the main text. *N* = 5202 sperm cells, 19 sand martin males, 11 barn swallow males with five males used twice for 35 experiments total; 21 barn swallow females with two used twice, 23 sand martin females

Parameter	*F*_df_ (*P* value)
Time	*F*_1,5180.8_ = 21.35 (<0.001)
Male species	*F*_1,29.6_ = 0.14 (0.71)
Treatment	*F*_2,95.3_ = 4.06 (0.02)
Male species × Time	*F*_1,5176.9_ = 8.33 (0.004)
Treatment × Time	*F*_2,4126.5_ = 3.31 (0.04)

Similarly, the proportion of motile cells decreased over time in both species of swallows, with motility depending on male species and treatment (Fig. 2C,D; Table 3). A higher proportion of cells were motile in female fluids compared to control (estimated difference = 0.07 ± 0.03 for each female species relative to control; both *t* > 2.35, adjusted *P* < 0.02; difference between female treatments = 0.00 ± 0.03, *t* = 0.01, *P* = 0.99). The proportion of motile sperm decreased faster in sand martin ejaculates (estimated change over time: −0.09 ± 0.02) compared to barn swallow ejaculates (0.00 ± 0.02). Interaction terms that were predicted in the presence of a postcopulatory prezygotic barrier were nonsignificant and removed from the model (male species × treatment × time, *F*_2,329.75_ = 1.0903, *P* = 0.34; male species × treatment, *F*_2,46.53_ = 0.41, *P* = 0.66).

**Table 3:** Reduced model relating the proportion of motile cells to treatments and time in swallows, after removing nonsignificant interaction terms. Post hoc comparisons between treatment groups are reported in the main text. *N* = 403 filming locations, 11 barn swallow males, 22 sand martin males, 22 barn swallow females, and 24 sand martin females, with two barn swallow females used twice and five barn swallow males used twice

Parameter	*F*_df_ (*P* value)
Time	*F*_1,296.2_ = 7.29 (0.01)
Male species	*F*_1,36.3_ = 3 (0.09)
Treatment	*F*_2,75.5_ = 3.81 (0.03)
Male species × Time	*F*_1,308.8_ = 7.87 (0.01)

Males varied substantially in both sperm swimming velocity and the proportion of motile cells, as indicated by the high proportion of model variance that was attributable to the random effect of male identity (Table 4).

**Table 4:** Percentages of model variance attributable to random effects of male and female identity (ID), the interaction between identities, and year, for sperm behavior of three pairs of taxa. The dependent variable was sperm velocity (VCL) or the proportion of motile cells (PM)

	Swallows		Bluethroats	Tits	
	VCL	PM	VCL	VCL	PM
Male ID	17.8	39.3	11.5	8.8	39.5
Female ID	0.2	0.0	3.6	0.3	6.9
Male:Female ID	3.0	19.6	0.0	4.4	12.3
Year	23.9	14.0	0.0	NA	NA

Note that for bluethroats, proportion motile could not be analyzed with generalized linear mixed models and so that response variable is not included here (NA, not applicable).

### Bluethroats

**Figure 3:**
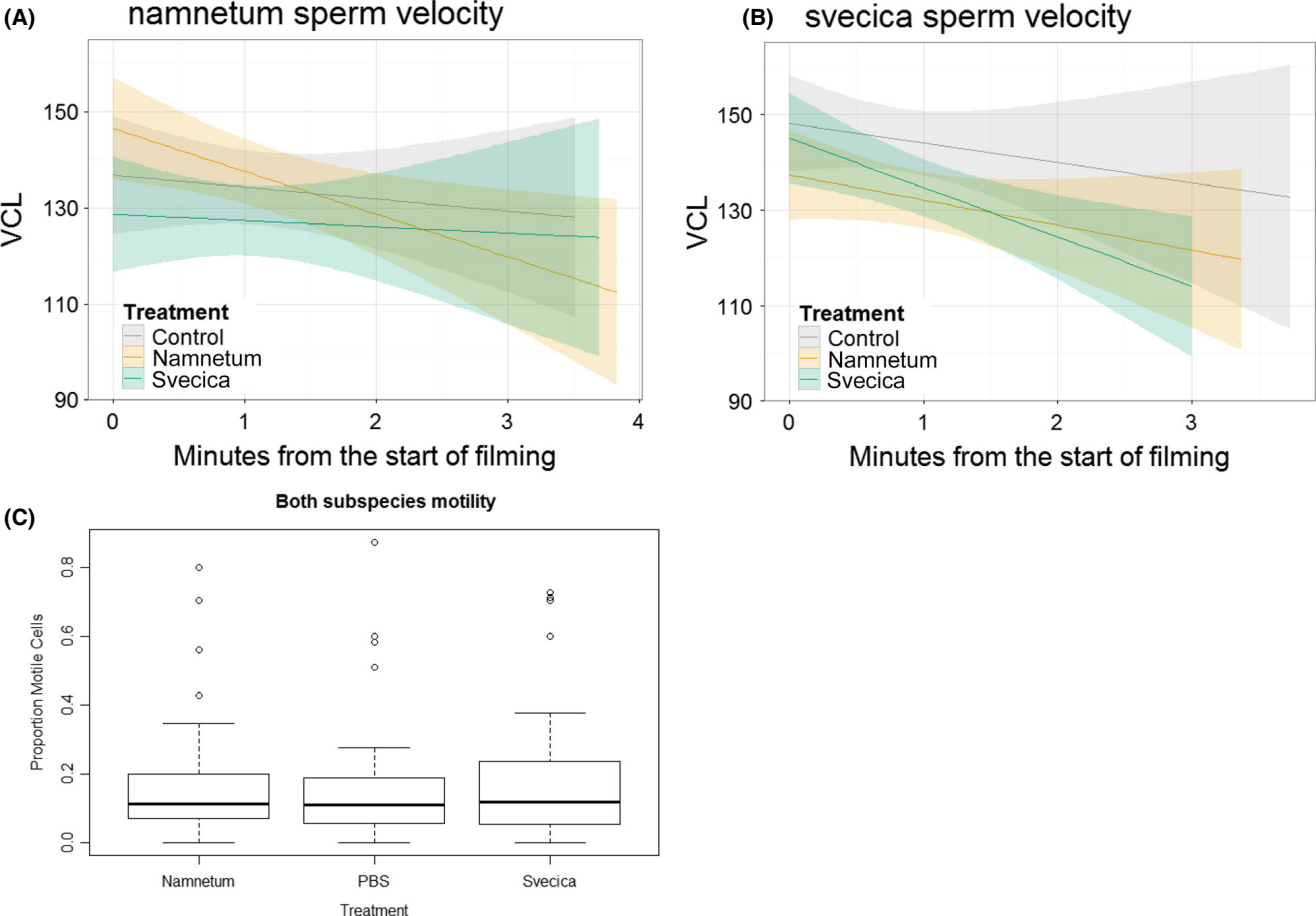
Effect of experimental treatments on sperm swimming parameters for two subspecies of bluethroats (A: *L. s. namnetum*; B: *L. s. svecica*; C: both subspecies). A, B: sperm velocity was measured over time fluid samples from the reproductive tract of *namnetum* females (tan) and *svecica* females (blue), or control (gray). Sperm velocity decreased over time, but this decrease was not related to either male or female subspecies. (C) The proportion of motile cells was measured in conspecific and heterospecific female fluids and in control saline solution (PBS), and could not be analyzed with respect to time because of the distribution of the data. Plots A and B drawn using ggplot2 using raw data; shading indicates 95% confidence intervals; statistical tests were performed with both male species considered simultaneously, although they are drawn separately here.

Curvilinear velocity (VCL) decreased over time in bluethroats similarly in both treatments and for both male species (Fig. 3A,B; Table 5). Interaction terms that were predicted in the presence of a postcopulatory prezygotic barrier were nonsignificant and removed from the model (male species × treatment × time, *F*_2,1479.1_ = 1.03, *P* = 0.36; male species × treatment, *F*_2,5.73_ = 0.52, *P* = 0.62). Males varied substantially in the swimming speed of their sperm (Table 4). The proportion of motile cells did not differ significantly between any pair of comparisons (all Wilcoxon V > 269, *P* > 0.25, Fig. 3C).

**Table 5:** Reduced model relating sperm velocity to treatments and time in bluethroats, after removing nonsignificant interaction terms. *N* = 1594 cells, *n* = 10 *namnetum* males and 13 *svecica* males; 14 *namnetum* and 15 *svecica* females

Parameter	*F*_df_ (*P* value)
Time	*F*_1,1587.6_ = 15.29 (<0.001)
Male species	*F*_1,18.0_ = 0.01 (0.93)
Treatment	*F*_2,21.5_ = 0.22 (0.80)

### Tits

**Figure 4:**
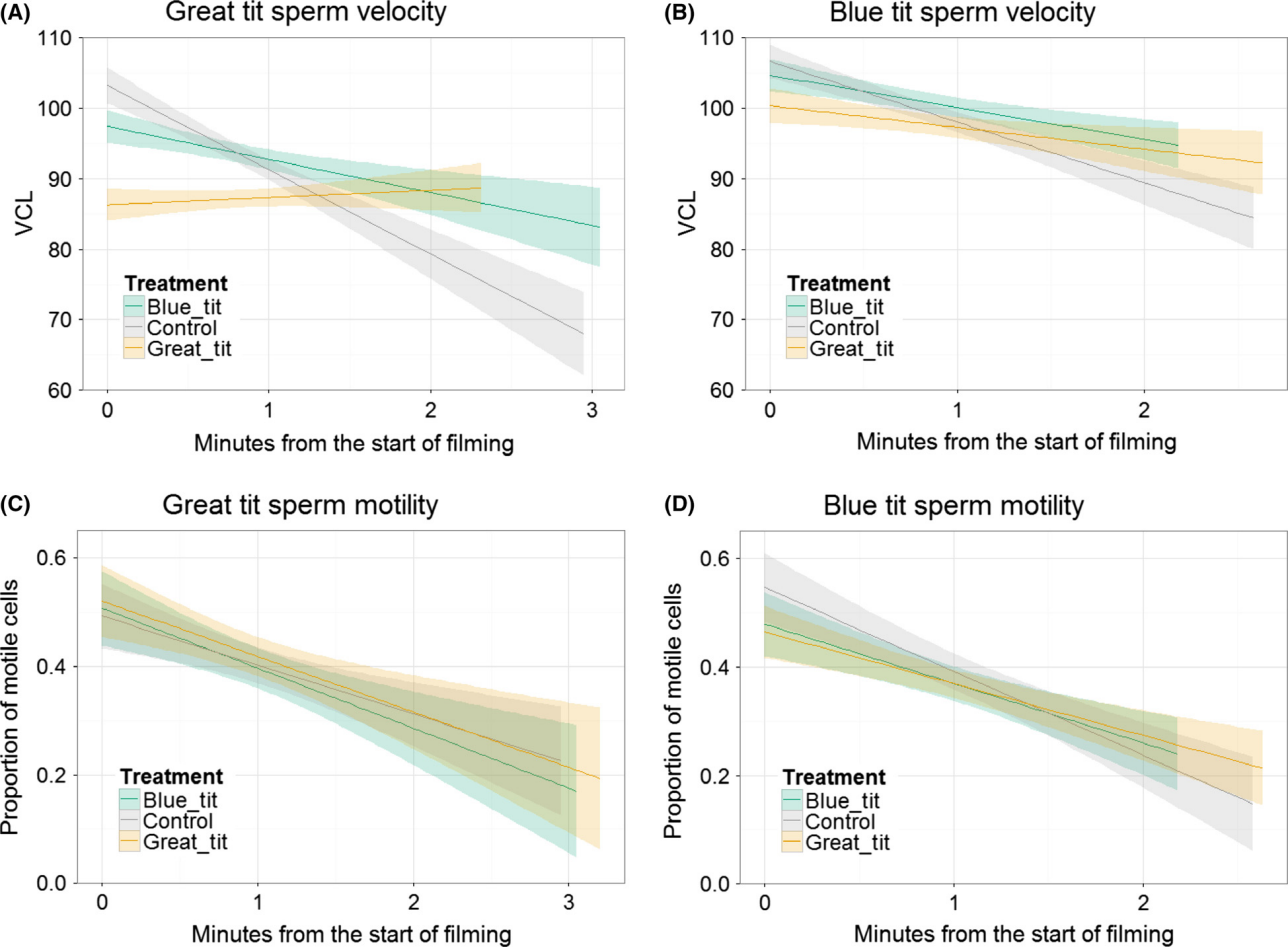
Change in sperm swimming parameters (A, B: sperm velocity, VCL,* μ*m/S; C, D: proportion of motile sperm) over time in great tits (A, C) and blue tits (B, D). Sperm were mixed with fluid from the reproductive tract of blue tit females (blue), great tit females (tan) or a control saline solution (gray) before being filmed. (A, B) For sperm of both species, swimming speed decreased more quickly in the control treatment than in either female fluid. The initial swimming speed of sperm was lower in great tit female fluid than in other treatments. (C, D) Proportion of motile sperm decreased over time, with the decrease being faster in blue tit males than great tit males. Plots drawn using ggplot2 using raw data; shading indicates 95% confidence intervals; statistical tests were performed with both male species considered simultaneously, although they are drawn separately here.

Blue tit and great tit sperm velocity decreased more over time in control fluids than in female fluids from either species (Fig. 4A,B; Table 6). The estimated difference in slope for blue tit females relative to control was 5.12 ± 1.26 (*t*_8165_ = 4.02; adjusted *P* < 0.001), for great tit females relative to control was 6.70 ± 1.24 (*t*_8209_ = 5.41, adjusted *P* < 0.001), and the difference in slopes between the female species was not significant (−1.57 ± 1.12, *t*_8205_ = −1.40, adjusted *P* = 0.17). Female great tits also reduced estimated initial sperm swimming speed (i.e., the intercept) by 5.57 ± 2.10 (*t*_39_ = −2.65, adjusted *P* = 0.02) compared to blue tit females, and by 7.95 ± 2.16 (*t*_38_ = 3.678, adjusted *P* = 0.002) compared to control; the difference in intercept between blue tit females and control was not significant (estimated difference 2.38 ± 2.18, *t*_39_ = 1.20, adjusted *P* = 0.28). Interaction terms that were predicted in the presence of a postcopulatory prezygotic barrier were nonsignificant and removed from the model (male species × treatment × time, *F*_2,8196.9_ = 0.40, *P* = 0.67; male species × treatment, *F*_2,23.4_ = 0.21, *P* = 0.81).

**Table 6:** Reduced model relating sperm velocity to treatments and time in tits, after removing nonsignificant interaction terms. Post hoc comparisons between treatment groups are reported in the main text. *N* = 8252 sperm cells, 25 experiments, 12 blue tit males, and 13 great tit males, with 13 females of each species

Parameter	*F*_df_ (*P* value)
Time	*F*_1,8237.9_ = 88.95 (<0.001)
Male species	*F*_1,21.9_ = 3.44 (0.08)
Treatment	*F*_2,38.3_ = 7.28 (0.002)
Treatment × Time	*F*_2,8195.8_ = 15.1 (<0.001)

The proportion of motile sperm decreased over time in tits (Fig. 4C,D; Table 7), with the decrease being faster in blue tit males than great tit males (estimated difference in slope, −0.04 ± 0.01). Interaction terms that were predicted in the presence of a postcopulatory prezygotic barrier were nonsignificant and removed from the model (male species × treatment × time, *F*_2,541.84_ = 1.49, *P* = 0.23; male species × treatment, *F*_2,21.3_ = 0.41, *P* = 0.67).

**Table 7:** Reduced model relating the proportion of motile sperm to treatments and time in tits, after removing nonsignificant interaction terms. Post hoc comparisons between treatment groups are reported in the main text. *N* = 600 filming locations, 25 experiments, 12 blue tit males, and 13 great tit males, with 13 females of each species

Parameter	*F*_df_ (*P* value)
Time	*F*_1,528.2_ = 125.97 (<0.001)
Male species	*F*_1,26.0_ = 0.01 (0.91)
Treatment	*F*_2,22.4_ = 0.27 (0.77)
Male species × Time	*F*_1,528.0_ = 6.03 (0.01)

Males varied substantially in the proportion of motile cells, and to a lesser degree in the velocity of their sperm (Table 4).

### Proteins

Sand martin female fluid samples had lower total protein concentration (*F*_1,28.0_ = 17.94, *P* = 0.0002) and fewer detected bands (*F*_1,28_ = 21.48, *P* < 0.0001) than did barn swallow female fluid samples (Table 8). There was no significant difference in measured protein parameters depending on storage duration for the swallows (*P* > 0.25 for both parameters; Table 8). Parameters for bluethroat female fluids did not differ between subspecies or years (*P* > 0.4; Table 8). No excess fluid samples were available from bluethroats from 2012, due to logistics, for protein analysis. Parameters for tit females did not differ between species (*F* < 1, *P* > 0.3; Table 8). We did not attempt to relate protein concentrations from individual females to their breeding stage, age, or other potential explanatory factors because we lacked detailed information on breeding stage for most individual females, and because sample sizes were small. However, in preliminary investigations, we did not find that differences in VCL or the proportion of motile cells among treatments within an ejaculate depended on the concentration of proteins in the female fluids the ejaculate was exposed to, for any of the study species (data not shown).

**Table 8:** Protein content (mean ± SD) of female fluid samples from different species. Swallow and bluethroat samples were analyzed in October 2014 or February 2015, so that the year of sampling closely reflects storage duration. Tit samples were analyzed in August 2015. Raw averages are given here; log transformation was applied before statistical analysis

Female species	Year sampled (*n* females tested)	Total protein concentration	# bands detected
Barn swallow	2013 (7)	269.7 ± 268.2	12.3 ± 4.1
	2014 (9)	94.9 ± 57.2	10.4 ± 2.5
Sand martin	2013 (7)	47.2 ± 39.4	6.1 ± 4.3
	2014 (8)	26.9 ± 23.5	5.1 ± 3.0
Bluethroat (ssp. *namnetum*)	2013 (5)	202.1 ± 99.9	9.0 ± 3.3
	2014 (3)	341.3 ± 31.6	11.0 ± 3.0
Bluethroat (ssp. *svecica*)	2013 (3)	128.4 ± 16.6	12.7 ± 0.7
	2014 (3)	105.3 ± 65.2	10.0 ± 2.0
Blue Tit	2015 (5)	582. 6 ± 558.5	13.8 ± 2.77
Great Tit	2015 (5)	663.7 ± 610.5	18.0 ± 9.03

## Discussion

In three reciprocal crosses representing three taxonomic families, we found no evidence of females discriminating against heterospecific sperm. While females of different species had differing effects on sperm swimming speed, these effects did not depend on the species of the male, as would need to be the case for a postcopulatory prezygotic barrier to be in action. Similarly, sperm from different male species showed different performance over time, but these differences were not linked to the species of the female fluid in which they swam.

Our tests simulated sperm swimming across the vagina immediately after copulation, which has been identified as the most likely stage between copulation and fertilization for postcopulatory prezygotic barriers to arise in passerines (Birkhead and Brillard 2007). Our results are therefore an important contribution to our understanding of species boundaries in this group, suggesting that for species that do not routinely interbreed in nature (Table 1), reduced sperm performance while sperm swim across the vagina is unlikely to be a common reproductive barrier. Such reproductive barriers may, however, be important when females have previously been exposed to heterospecific sperm (E. R. A. Cramer, M. Ålund, S. E. McFarlane, A. Johnsen, A. Qvarnström, unpublished data), and theoretical models suggest that reinforcement‐like processes can drive the adaptive evolution of such barriers in interbreeding species (Lorch and Servedio 2007). For noninterbreeding species, other postcopulatory prezygotic barriers are also possible, as outlined in the introduction; these could include, for instance, reduced heterospecific fertilization success due to incompatible sperm surface proteins (e.g., Zigler et al. 2005).

We chose our study species pairs in part because their sperm lengths have diverged substantially, and we assumed that this length divergence might also indicate divergence in traits such as sperm surface protein complement, which we hypothesize to be the primary mechanism of female choice while sperm swim across the vagina (following Steele and Wishart 1992, 1996). That assumption remains to be tested. Sperm length itself may be more important as a possible reproductive barrier during sperm storage in the female sperm storage tubules (Briskie et al. 1997), that is, at a later stage between copulation and fertilization. In passerines studied to date, sperm total length differs by 3.8 ± 4.0% (mean ± SD; range 0.3–11.6%) among subspecies and by 3.5 ± 4.4% (range: 0.3–9.9%) between sister species (reviewed in Hogner et al. 2013). The degree of divergence between the species we studied here is of a similar scale, suggesting that our results should be relevant for addressing the role of diverged sperm morphology as an isolating mechanism between incipient species. High divergence in sperm length among bluethroat subspecies (Hogner et al. 2013; Dobbe 2014) suggests that sufficient time has passed for postcopulatory prezygotic barriers to have arisen between these subspecies, despite their recent divergence. Postcopulatory prezygotic barriers, and particularly reproductive proteins, evolve quite rapidly in other systems (Pitnick et al. 2003; Dorus et al. 2004; Ramm et al. 2009). It should also be noted that, while diverged sperm morphology between species does not appear to indicate an inability of sperm to function in a heterospecific female environment, sperm morphological divergence can provide insight into the historical levels of gene flow between species (Gohli et al. 2015). That is, sperm morphology has a strong genetic basis (Mossman et al. 2009), so if there had been high levels of interbreeding between our taxa in the recent past, we might expect populations to have similar, rather than diverged, sperm morphology.

It is also noteworthy that the degree of interspecific divergence in precopulatory phenotypes does not strictly correlate with the resistance of species to interbreeding (e.g., Runemark et al. 2011; see also Maan and Seehausen 2012; Rodríguez et al. 2013). The frequent occurrence of asymmetrical reproductive isolating barriers (Coyne and Orr 2004) within pairs of species – where the strength of the reproductive barrier depends on the direction of the cross, but the phenotypic difference does not – further demonstrates that the relationship between trait divergence and reproductive isolation is complex. Simple divergence in reproductive traits between species should therefore not be taken as a strict indicator of the likelihood of a reproductive barrier between them, whether the trait under study is involved in precopulatory mate choice or postcopulatory prezygotic processes.

The proportion of motile sperm cells decreased strongly over time in all species tested (although we were unable to test this statistically in bluethroats). Sperm swimming speed also declined in both tit species and in both bluethroat subspecies, but it was fairly constant in barn swallows and increasing in sand martins (although over a longer time scale, sand martin sperm swimming speed also declines; Helfenstein et al. 2008). It is intuitively appealing to attribute the reductions in sperm swimming speed and the proportion of motile sperm over time to sperm dying or exhausting their energy reserves and ceasing to swim; variation among species could potentially be linked to variation in sperm metabolic pathways (Wishart 1982; Cummins 2009). It is unclear whether that is the case. It is also intriguing to note that changes in sperm swimming speed and motility are not necessarily parallel; for example, the direction of change differs for sand martin males, and the responsiveness of the two measures to the three different treatments also differed in the tit and swallow datasets. Differences in how treatments affect sperm velocity, compared to the proportion of motile cells, have also been documented in poultry (Wishart and Wilson 1999; Froman and Kirby 2005). For both measures of sperm performance, we observed substantial among‐male variation, as a large proportion of the variation in sperm performance could be attributed to the random effect of male identity. This result suggests that a male's sperm performance in the three treatments are correlated, which in turn may imply that selection for improved sperm performance in conspecific females can cause improved sperm performance also in a heterospecific environment. Further work investigating the repeatability of sperm performance across different sampling events (e.g., Cramer et al. 2015), as well as investigating heritability, would be needed to understand how sperm performance responds to selective pressures.

In both swallows and tits, female fluid from either female species improved sperm performance relative to the control (increasing the proportion of motile cells in swallows, and helping to maintain a high VCL in tits). We suggest that females may provide metabolic substrates to sperm cells, or that other compounds in the female fluids alter sperm behavior. In both *Drosophila* (Lüpold et al. 2012) and poultry (Pizzari et al. 2007), sperm behavior can be altered by mixing sperm from one male with seminal fluid from other males, demonstrating that sperm are capable of responding to such exogenously derived compounds.

Møller et al. (2008) found that barn swallow female fluid slowed barn swallow sperm relative to their control medium, while we found that female fluid increased the proportion of motile sperm and increased sperm velocity over time. Differences in experimental design and analytical approach make it difficult to directly compare these studies; in particular, Møller et al. (2008) used a different solution for their control medium, and they collected female fluids using a slightly different procedure. Furthermore, the sperm performance metric used by Møller et al. (2008) contained information about both sperm velocity and the proportion of motile sperm simultaneously, whereas we have analyzed them separately.

We observed substantial variation among females within species in total protein concentration and in the number of protein peaks detected. We hypothesize that variation may be partially explained by variation in when we sampled female fluids relative to egg laying. Other factors such as variation in cloacal morphology and female hydration may also have affected our ability to sample reproductive tract fluid. While it would be interesting to relate the effect a female's fluid sample had on sperm behavior to the proteins present in that sample, conducting such tests would require proteomic analyses that are beyond the scope of this study.

In conclusion, we suggest that postcopulatory prezygotic barriers acting on sperm as they swim across the vagina are unlikely to represent a widespread reproductive barrier in passerine species that do not routinely hybridize with each other. Evolutionary theory suggests that postcopulatory prezygotic barriers can evolve via a reinforcement‐like process in hybridizing species (Lorch and Servedio 2007), and previous exposure to heterospecific sperm appears to contribute to the development of a postcopulatory prezygotic barrier in *Ficedula* flycatchers (E. R. A. Cramer, M. Ålund, S. E. McFarlane, A. Johnsen, A. Qvarnström, unpublished data). Barriers at this stage between copulation and fertilization may therefore be important under certain conditions. However, the absence of apparent barriers between copulation and fertilization in all three of the species pairs tested here, as well as in a fourth species pair from another taxonomic family (Cramer et al. 2014), suggests that such barriers are uncommon for populations that do not currently hybridize.

## Conflict of Interest

None declared.

## Appendix 1

### Summary of experiments and methods used in testing a postcopulatory prezygotic barrier between swallow species, bluethroat subspecies, and tit species. Each treatment was filmed in multiple locations (# filmings), with either a HDR‐HC1E Sony camera or a Legria HF S200 Cannon camera, attached to an Olympus CX41 microscope; total magnification (Mag) was adjusted using zoom settings on the camera. Microscope slides were maintained at 40°C with either a Hamilton Thorne MiniTherm stage heater (HT) or a Tokai Hit TP‐S glass stage (TP‐S).

**Table:**

Taxon	Year (*n* experiments^a^)	Breeding period of females	# filmings	Camera	Mag.	Stage heater
Barn swallows	2013 (*n* = 11, 7, 7)	Incubating to nestlings	3	Cannon	400	HT
	2014 (*n* = 8, 8, 8)		4	Cannon	320	TP‐S
Sand martins	2013 (*n* = 11, 10, 10)	Laying to incubating	3	Cannon	400	HT
	2014 (*n* = 13, 9, 12)		4	Cannon	320	TP‐S
*L. s. svecica*	2012 (NA)^b^	Mid‐incubation	NA	NA	NA	NA
	2013 (*n* = 15, 6, 14)	Prelaying to late laying	8	Sony	400	HT
	2014 (*n* = 3, 3, 3)	Nest‐building	8	Sony	215	TP‐S
	2015 (*n* = 5, 4, 5)	Prebuilding	8	Sony	215	TP‐S
*L. s. namnetum*	2013 (*n* = 6, 2, 3)	Prelaying	4	Sony	400	HT
	2014 (*n* = 12, 7, 11)	Prelaying	8	Sony	225	TP‐S
	2015 (*n* = 3, 1, 3)	Prelaying	8	Sony	225	TP‐S
Blue tits	2015 (*n* = 12, 12, 12)	Laying	8	Sony	400	TP‐S
Great tits	2015 (*n* = 13, 13, 13)	Laying, one building	8	Sony	400	TP‐S

NA = not applicable.

a *N* experiments performed, *N* experiments included in analyses of VCL over time, and *N* experiments included in analyses of proportion motile; see “Statistical analysis” section for information on criteria for experiments to be included.

b Experiments were not conducted on *L. s. svecica* in 2012.
